# Long-Term Trends and Prognosis in Cardiovascular Mortality in the Kazakhstani Population Living Around the Semipalatinsk Nuclear Test Site

**DOI:** 10.3390/ijerph23070874

**Published:** 2026-07-05

**Authors:** Dariya Shabdarbayeva, Lyudmila Pivina, Nailya Chaizhunussova, Andrey Orekhov, Galiya Alibayeva, Meruyert Massabayeva, Assel Baibussinova, Gulnara Batenova, Zhanargul Smailova, Saulesh Apbassova, Saule Kozhanova, Madina Abenova, Alexandra Lipikhina, Asset Izdenov, Diana Ygiyeva, Raushan Dosmagambetova, Altay Dyussupov

**Affiliations:** 1 Office of the Vice Rector, Semey Medical University, Semey 071400, Kazakhstan; dariya.shabdarbaeva@smu.edu.kz (D.S.);; 2 Department of Emergency Medicine, Semey Medical University, Semey 071400, Kazakhstan; 3 Department of Public Health, Semey Medical University, Semey 071400, Kazakhstan; 4 Department of Internal Medicine, Semey Medical University, Semey 071400, Kazakhstan; 5 Research Laboratory Center, Semey Medical University, Semey 071400, Kazakhstan; 6 Department of Biostatistics, Semey Medical University, Semey 071400, Kazakhstan; 7 Department of Pathology, Semey Medical University, Semey 071400, Kazakhstan; 8 Department of Anatomy, Semey Medical University, Semey 071400, Kazakhstan; 9 Research Institute of Radiation Medicine and Ecology, Semey Medical University, Semey 071400, Kazakhstan; 10 Ministry of Health of the Republic of Kazakhstan, Astana 010000, Kazakhstan; 11 Office of the Rector, Semey Medical University, Semey, 071400, Kazakhstan

**Keywords:** cardiovascular diseases, mortality rate, years of life lost, radiation hazards, radioactivity, Semipalatinsk nuclear test site

## Abstract

**Highlights:**

**Public Health Relevance—How does this work relate to a public health issue?**

- Nuclear tests at the former Semipalatinsk Nuclear Test Site were conducted over a period of 40 years, resulting in significant radioactive contamination of surrounding territories and affecting the population.
- Cardiovascular diseases remain a major cause of mortality in affected populations, highlighting the importance of evaluating their long-term health consequences.

**Public Health Significance—Why is this work of significance to public health?**

- Forecasting of cardiovascular disease indicators demonstrates a continuing upward trend among the exposed population.
- The study identified a significant association between radiation dose and cardiovascular mortality, with elevated years of life lost for several major cardiovascular conditions.

**Public Health Implications—What are the key implications or messages for practitioners, policy makers and/or researchers in public health?**

- The results confirm the need for further research aimed at studying the effects of ionizing radiation on the descendants of directly exposed individuals.
- The findings support the need for continued public health surveillance and further research on the long-term cardiovascular consequences of environmental radiation exposure.

**Abstract:**

**Background:** The purpose of the study is the assessment of mortality from cardiovascular diseases (CVDs) and their dose–response relationships and the calculation of the number of years of life lost (YLL) in Kazakhstani residents living in territories around the Semipalatinsk nuclear test site. **Materials and Methods:** The study is based on the State Scientific Automated Medical Registry (SSAMR) database. The study included 3482 residents of the Abay and Beskaragai districts exposed to radiation and 1886 residents of the Kokpekty district (control group). The median equivalent radiation dose for the exposed group was 864.0 mSv, compared to 64.4 mSv in the control group. The study period was from 1949 to 2024. **Results:** Mortality rates in the exposed group exceeded those of the comparison group throughout the study. The relative risk (RR) of mortality was 1.41 for all CVDs, 2.0 for stroke, 7.88 for chronic cerebrovascular disease (CCVD), and 2.39 for congenital heart disease (CHD). Age-standardized mortality rates were higher in the radiation-exposed population, with the highest excess risk recorded in 1960–1964 (RR = 5.31; 95% CI 4.32–6.53). The number of YLL from acute myocardial infarction (AMI) was 6097.0 in the exposed group versus 5893.0 in the comparison group, 3857.5 from hemorrhagic stroke versus 1996.9, and 2696.6 from CHD versus 957.7. An increase in radiation dose by 1 cSv was associated with an 8.5% increase in the odds of death from CVDs (OR = 1.085; 95% CI 1.075–1.094; *p* < 0.001). Radiation dose demonstrated good predictive ability for mortality from cardiovascular diseases (AUC = 0.700). **Conclusions:** The results indicate an increased risk of CVD mortality among residents of radiation-contaminated areas of Kazakhstan throughout the study period.

## 1. Introduction

Assessing the relationship between exposure to low- and medium-dose ionizing radiation and the development of cardiovascular diseases (CVDs) is one of the most pressing issues in modern radiation medicine. It is well known that CVDs remain the leading cause of disability and mortality worldwide, despite advances in modern cardiology services [1,2]. Radiation exposure is one of the additional risk factors for CVD development [3,4]. The impact of radiation on the human body can be associated with medical and diagnostic procedures, radiation accidents, industrial conditions, and nuclear weapons testing [5]. The first studies to assess the association between radiation exposure and excess risks of cardiovascular morbidity and mortality were conducted in cohorts of Japanese people who survived the atomic bombing [6,7,8,9]. Increased risks of mortality from CVDs were found among groups of people exposed to radiation in occupational settings [10,11].

The former Semipalatinsk Nuclear Test Site (SNTS) is located in northeastern Kazakhstan, in a steppe and semi-desert zone, covering an area of 18,500 square kilometers. Between 1949 and 1989, 458 nuclear charges of varying yields were tested at the SNTS, including 148 ground and air explosions, until 1963, when ground tests were banned. Between 1949 and 1956, the main dose-generating explosions were conducted, leaving localized radioactive traces with a relatively high degree of contamination of the surrounding environment with biologically hazardous radionuclides. This led to the formation of a unique radiation situation, which resulted in long-term combined acute external exposure from gamma radiation and chronic internal exposure from the intake of radionuclides from the environment to large populations in the areas adjacent to the test site, in the range of low and medium doses [12,13].

Studying the health of Kazakhstani residents exposed to radiation due to the activities of the nuclear test site began in 1957, when a specialized institution, Dispensary No. 4, was established. Its tasks included monitoring the environmental situation in the areas surrounding the test site and screening the population of these areas, including physical examinations, laboratory tests, and instrumental examinations. However, the research was very limited due to the high level of secrecy surrounding information about the radiation situation [14]. Only after the end of nuclear testing in 1989 did staff of the Research Institute of Radiation Medicine and Ecology, the successor to Dispensary No. 4, begin large-scale studies of the health of the radiation-exposed population, in collaboration with foreign scientists. Since 2002, the State Scientific Automated Medical Registry of Population Exposed to Radiation (SSAMR) has been compiled, serving as a basis for studying radiation effects [15,16]. However, very few studies have been conducted on cardiovascular problems, making it difficult to assess their relationship with radiation exposure. Research in cardiology has focused primarily on assessing the incidence and prevalence of specific CVDs, such as hypertension and stroke [17,18]. Only two studies have attempted to examine the effect of ionizing radiation on cardiovascular mortality [19,20].

In our previous study, the results of which were published in 2025, we attempted to estimate the structure of CVD mortality and the dose relationship between exposure to ionizing radiation and the risk of mortality from major CVDs among the population of Kazakhstan living in areas adjacent to the SNTS [20].

The aim of this study was to assess the dynamics and forecast mortality rates, as well as to calculate the number of years of life lost (YLL) due to CVDs in residents of Kazakhstan living in areas exposed to radioactive contamination.

## 2. Materials and Methods

### 2.1. Information About the State Scientific Automated Medical Registry

Our cross-sectional study is based on a subregister of mortality of residents of areas adjacent to the test site. This subregister is part of the SSAMR. Its creation began in 2002 at the initiative of the Ministry of Health of the Republic of Kazakhstan with the assistance of Japanese scientists from the Radiation Effect Research Foundation [15] for the purpose of registration, collection of demographic and medical information and monitoring of the health status of residents of Kazakhstan related to radiation situations of past years due to the activities of the SNTS. Furthermore, the registry contains information on the health status of not only individuals directly exposed to radiation but also their second- and third-generation descendants. In total, the registry database contains information on over 370,000 citizens of Kazakhstan. Each registry member has their own unique identification number (Sysid), allowing access to all relevant information contained in the database [21].

The mortality subregistry contains data on 5368 individuals who died from CVDs. For each member of the subregistry, information is available on the date of birth and death, age, gender, ethnicity, death code according to the International Classification of Diseases, 10th Revision (ICD-10), place of permanent residence, and radiation dose in Sievert (Sv). Causes of death were coded by medical professionals. The subregistry contains information from 1949 to 2024. It was copied from the regional archive of the civil registry office (ZAGS) and verified in the Integrated Medical Information System of the Republic of Kazakhstan and the akimats (governing bodies) of the settlements where the deceased citizens resided. The main source of information was death certificates completed by the attending physician and having the status of an official document.

Information on the population data of the studied areas was obtained from the National Statistical Bureau of Kazakhstan, as well as from archival data.

### 2.2. Characteristics of the Studied Population

The study (exposed) group included 3482 residents of radiation-exposed settlements in the Abaysky District (the villages of Karaul, Sarzhal, and Kainar) and the Beskaragaysky District (the villages of Dolon, Mostik, and Cheremushka). A total of 1886 residents of the Kokpekty District, where the radiation dose was minimal, were included in the control (unexposed) group. All study participants died from cardiovascular diseases at different periods.

In the study group, 1873 people (53.4%) were male, while in the control group, 975 people (51.7%) were male. In the study group, 2823 (81.1%) participants were of Asian ethnicity, while in the control group, 1318 individuals were of Asian ethnicity (69.9%). The other study participants were of European ethnicity. The majority of individuals in both groups had secondary education and were engaged in agricultural activities; none of them worked in the SNTS area.

The study was approved by the Ethics Committee of Semey Medical University (protocol No. 2b; 18 December 2024).

### 2.3. Assessment of Radiation Doses for Individuals Included in the Study

The radiation doses of the studied population were calculated using a specially created program included in SSAMR, in accordance with the official methodological recommendations adopted in the Republic of Kazakhstan [22]. The radiation dose calculation is based on parameters such as the yield of the atmospheric and underground explosions, the altitude of the detonation, wind speed, climatic conditions at the time of the test, the location of the radioactive cloud trace, and the duration of residence in the contaminated area. This methodology is based on a joint US-Russian dose reconstruction methodology, developed based on the combined experience of dose reconstruction experts [23,24].

Radioactive fallout due to the activity of SNTS consisted of a mixture of fission products and neutron-activated materials. The principal radiological hazards were associated with both long-lived and short-lived radionuclides, including strontium-90 (^90Sr), cesium-137 (^137Cs), plutonium isotopes (^239Pu, ^240Pu), americium-241 (^241Am), and iodine-131 (^131I). The main characteristics of these radionuclides are presented in Table 1. In addition to these radionuclides, radioactive cloud plumes also contained radiocarbon (^14C), tritium (^3H), cobalt-60 (^60Co), and various isotopes of rare-earth elements. The relative contribution of individual radionuclides to the radiation dose depends strongly on the onset time of radioactive fallout and the duration of exposure [25,26].

According to the Methodological Recommendations of the Republic of Kazakhstan [22], the fundamental criterion for individual dose assessment is the determination of the contribution of both external gamma radiation exposure and internal radiation exposure resulting from the intake of long-lived radionuclides into the human body through inhalation of contaminated air during the passage of radioactive clouds, as well as through the consumption of contaminated drinking water and locally produced food products. Based on contemporary scientific evidence regarding the relative contributions of acute and chronic radiation exposure under conditions similar to those surrounding the Semipalatinsk Nuclear Test Site (SNTS), the following approach has been proposed for estimating cumulative radiation dose: external exposure is assumed to contribute 80% of the maximum possible dose, whereas internal exposure is assumed to contribute 20% of the maximum possible dose.

The maximum possible dose is defined as the highest population exposure dose established by the Law of the Republic of Kazakhstan [27] according to residence in a specific settlement affected by the activities of the SNTS. Calculation of individual radiation doses takes into account each participant’s date of birth, age-specific behavioral factors, occupation, and residential history, including the end date of residence in each settlement, corresponding either to migration from the settlement or to death.

Dose reconstruction included all settlements in which study participants resided during the period 1949–1990 and which were affected by radioactive fallout resulting from nuclear weapons testing at the SNTS. Periods of residence outside the affected territories, including residence in remote regions of Kazakhstan or abroad, were not included in dose estimation because these locations were not affected by radioactive fallout from nuclear tests conducted at the SNTS.

The median equivalent dose for members of the study group was 864 mSv (IQR 604 mSv), while in the control group this value was 64.4 mSv (IQR 46.3 mSv).

Individual cumulative doses were calculated for the period from the beginning of radiation exposure (i.e., the arrival of radioactive fallout in the settlement of residence, the individual’s date of birth, or the date of arrival in a radiation-affected settlement) until the end of radiation exposure (i.e., cessation of nuclear testing, departure from the radiation-affected settlement, or death).

### 2.4. Methods of Statistical Analysis

Statistical analysis was performed using Jamovi (version 2.6), IBM SPSS Statistics version 26.0, and Python 3.9.12. Descriptive statistical methods were used to summarize the data. Continuous variables are presented as means and standard deviations (M ± SD) or medians and interquartile ranges (Me [Q1; Q3]), depending on the distribution of the data. Categorical variables are presented as absolute numbers and percentages.

To assess mortality, crude mortality rates (CMRs) were calculated as the number of deaths divided by the corresponding population size and expressed per 100,000 population. To eliminate the influence of differences in age structure between populations, age-standardized mortality rates (ASMRs) were additionally calculated using the direct standardization method based on the World Health Organization (WHO) World Standard Population [28]. Results are presented per 100,000 population.

To compare mortality rates between residents of radiation-exposed and comparison districts, relative risks (RRs) and corresponding 95% confidence intervals (95% CIs) were calculated. Differences were considered statistically significant when the 95% CI did not include 1.0. Comparisons of categorical variables were performed using Pearson’s χ^2^ test or Fisher’s exact test when expected cell frequencies were less than five.

Temporal mortality trends were analyzed using Joinpoint regression analysis (Jamovi (version 2.6), IBM SPSS Statistics version 26.0, and Python 3.9.12). Annual Percent Change (APC) and Average Annual Percent Change (AAPC) with 95% confidence intervals were calculated to quantify changes in mortality over time.

To assess premature mortality, Years of Life Lost (YLL) were calculated according to the methodology of the Global Burden of Disease (GBD) study. YLL estimates were based on the number of deaths in each age group and the corresponding standard life expectancy. Results are presented per 100,000 population.

Mortality forecasting through 2050 was performed using Autoregressive Integrated Moving Average (ARIMA) time-series models. The optimal model was selected based on the Akaike Information Criterion (AIC), Bayesian Information Criterion (BIC), and residual autocorrelation analysis. The estimated mean mortality rate was 600.0 per 100,000 population. The Akaike Information Criterion value was AIC = 202.04. The residual variance estimate (σ^2^) was 29.425, indicating moderate variability in the time series. Residual diagnostics demonstrated no statistically significant autocorrelation in either model. According to the Ljung–Box test, the *p*-value was 0.916 for the exposed group and 0.655 for the unexposed group, confirming the adequacy of the selected ARIMA models and the appropriateness of their use for forecasting CVD mortality. For all forecast estimates, 95% confidence intervals were calculated.

All statistical tests were two-sided, and differences were considered statistically significant at *p* < 0.05.

## 3. Results

### 3.1. Age-Standardized Mortality Rates from CVDs in Study Groups by Periods

Age-standardized mortality rates (ASMRs) from CVDs in the exposed group were higher than those in the controls throughout most of the observation period (Table 2). The maximum relative risks (RRs) were recorded in the period 1960–1964, when the ASMR in the study group was 2104.8 per 100,000 population (95% CI 1945.2–2264.5) versus 396.4 per 100,000 population (95% CI 320.2–472.6) in the control group. The RR in this period reached 5.31 (95% CI 4.32–6.53). High mortality rates were also observed in the periods 1954–1959 (RR = 3.43; 95% CI 2.71–4.33), 1965–1969 (RR = 3.42; 95% CI 2.87–4.08), and 1970–1974 (RR = 3.66; 95% CI 3.11–4.31).

Since the 1980s, a gradual decrease in differences between districts has been observed. Thus, in the period 1980–1984, the relative risk decreased to 1.42 (95% CI 1.26–1.60), and in the period 1990–1994, to 1.29 (95% CI 1.13–1.46). Despite the decrease in the effect size, the risk of death from CVDs in the exposed district continued to be statistically significantly elevated compared to the control district. After 2005, differences between districts disappeared.

**Figure 1:**
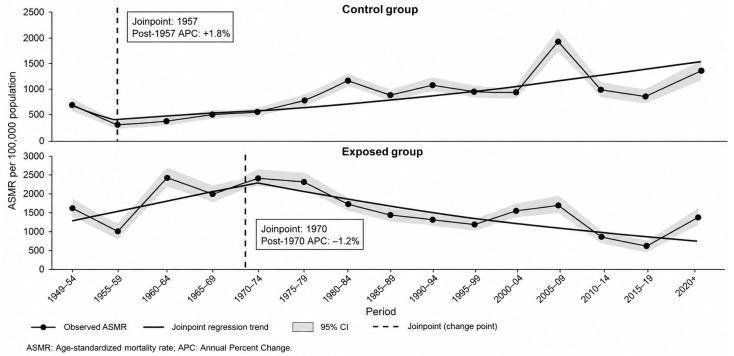
Trends in age-standardized CVD mortality rates.

Joinpoint analysis revealed a change in the long-term mortality trend in the study group before 1970 (1969.6; 95% CI 1953.9–1985.3). Before this period, an upward trend in mortality was observed (+31.5 cases per 100,000 population per year) (Figure 1). After the turning point, a decrease in mortality from CVDs was recorded at a rate of −17.6 cases per 100,000 population per year (95% CI from −29.9 to −5.4). Overall, this analysis is consistent with the RR estimate in the radiation-exposed group compared with the control group, with the most pronounced excess risk observed during the period 1960–1964, when the RR reached 5.31 (95% CI 4.32–6.53). Subsequently, a consistent decrease in the RR was observed (Figure 1).

### 3.2. Overall Mortality Rates Due to CVDs and Separate Diseases During the Study Period

Overall mortality from CVDs in the exposed group was significantly higher compared to the unexposed group: 612.9 per 100,000 population (95% CI: 592.7–633.6) versus 435.8 per 100,000 (95% CI: 416.3–455.9). The relative risk of death from CVDs in the exposed group was 1.41 (95% CI: 1.33–1.49) (Table 3). Relative risks (RRs) compare the exposed and unexposed groups and are presented with corresponding 95% CIs. An analysis of separate diseases revealed marked heterogeneity in intergroup differences in mortality rates. Thus, for the combined group of mortality from all strokes (ischemic and hemorrhagic), the relative risk of mortality was 2.00 (95% CI: 1.74–2.30). The most pronounced relative differences were observed for chronic cerebrovascular diseases (CCVDs), where mortality in the exposed group exceeded that of the control group by almost eight times: RR = 7.88 (95% CI: 4.98–12.48). For congenital heart defects (CHD), the relative risk of mortality was also quite high: RR = 2.39 (95% CI: 1.31–4.37). Only with respect to chronic ischemic heart disease (CIHD) did mortality rates in the control group exceed those in the exposed group: RR = 0.48 (95% CI: 0.43–0.52).

To compare the structure of cardiovascular mortality in individuals born during different periods of nuclear weapons testing at the SNTS, we divided them into a groups of people born before 1962, who were directly exposed to radiation during air and ground nuclear tests; people born from 1963 to 1989 (the cessation of ground nuclear weapons testing at the SNTS), when only chronic irradiation in the low-dose range was observed during radiation accidents with the release of radioactive gases to the surface; people born after 1990 were third-generation descendants of individuals exposed to radiation (they were not exposed to radiation, but continued to live in radiation-contaminated areas) (Table 4). In the main group, statistically significant differences in the structure of mortality from certain diseases were revealed in the subgroups of the study. After correction for multiple comparisons using the Benjamini–Hochberg method, significant differences remained for CIHD (*p* = 0.008); AMI (*p* = 0.005); and CHD (*p* < 0.001). While for CIHD and AMI there was a tendency toward a decrease in the proportion of deaths from these diseases depending on the date of birth of the exposed population, for CHD a sharp increase in the proportion of deaths was revealed in younger subgroups: from 0.2% among people born before 1962 to 15.8% among those born in 1963–1989 and 66.7% among those born after 1990 (*p* < 0.001). In the control group, after Benjamini–Hochberg correction, statistically significant differences between generations were identified for CIHD (*p* < 0.001), AMI (*p* < 0.001), CCVD (*p* < 0.001), and CHD (*p* < 0.001).

When comparing the mortality structure between the study and control groups, the most pronounced differences were found in CIHD, hemorrhagic stroke, and CCVD. Among people born before 1962, CCVD and hemorrhagic stroke were more frequently recorded in the study group, while the proportion of chronic ischemic heart disease was higher in the control group. Among individuals born between 1963 and 1989, the study group continued to have a higher proportion of deaths from CIHD and hemorrhagic stroke. It is noteworthy that in the study group, among individuals born after 1990, the mortality rate from CHD was statistically significantly higher than in the control group (Table 4).

### 3.3. Indicators of YLL Due to Mortality from Separate Types of CVDs in the Study Groups

The highest YLL rate was found for acute myocardial infarction (AMI), with it being statistically significantly higher in the exposed group than in the comparison group. The same trend was observed for CCVD, hemorrhagic stroke, and CHD. No statistically significant differences were found in the rates for hypertension between the comparison groups. For CIHD, the rate of life lost was statistically significantly higher in the comparison group than in the exposed group (Table 5).

### 3.4. Dynamics of Mortality Rates from CVDs and Their Separate Forms During the Study Period

**Figure 2:**
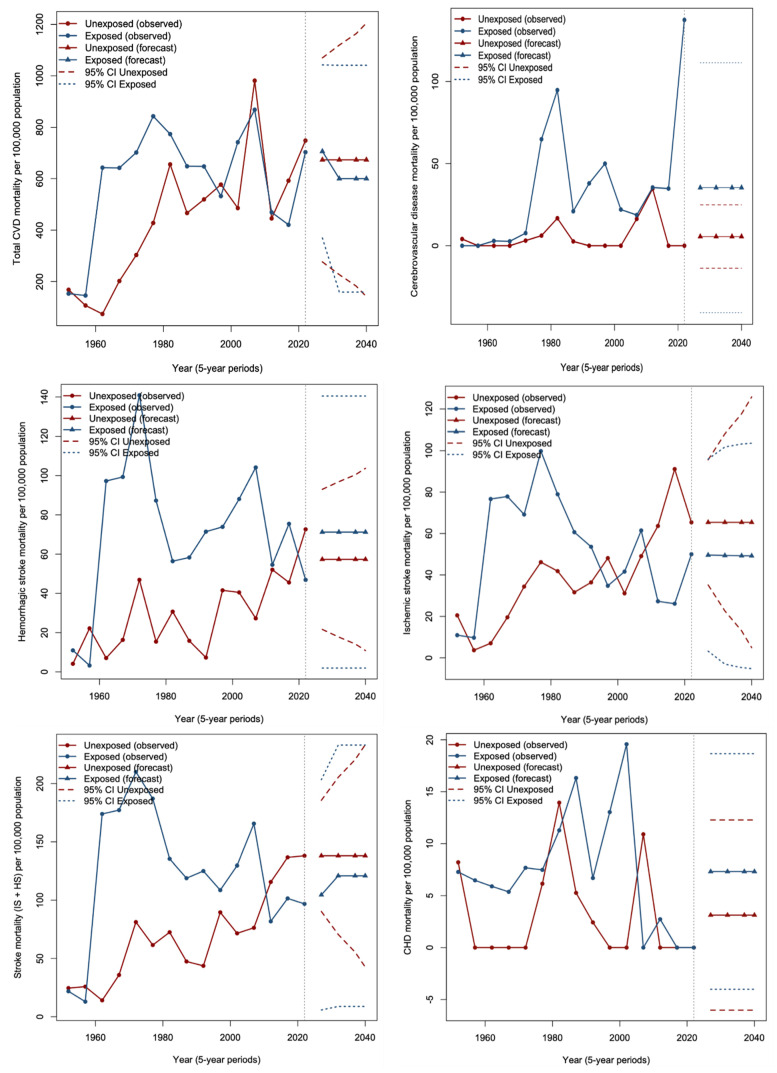
Dynamics of mortality rates due to separate CVDs and their forecasting until 2040.

In the exposed group, mortality from CVDs throughout the observation period was characterized by higher values compared to the comparison group. Already in 1962–1967, the mortality rate in the exposed group exceeded 640 per 100,000 population (642.8 and 641.7, respectively), with a subsequent increase to 843.1 in 1977. and 773.8 in 1982. In the later period, fluctuations in the indicator were observed; however, in 2007, the mortality again reached high values (868.3 per 100,000), and in 2022 it was 703.0 per 100,000 population. From 1962 until 1982, the differences maintained statistical significance (*p* < 0.001); in subsequent years, statistically significant differences were noted in separate time periods, such as 1987–1992, 2002 and 2017. The ARIMA-forecast indicates the preservation of the increased level of mortality from CVDs in the exposed group compared to the unexposed group until 2040 (Figure 2, Table S1).

Mortality from hypertension in both groups remained quite low during the entire observation period, which is typical for such nosology, which is rarely indicated as the main cause of death. However, the dynamic series was characterized by significant fluctuations, especially in the late period. ARIMA model was built for both groups, the adequacy of which was confirmed by the absence of autocorrelation in residuals (Ljung–Box test, *p* > 0.05). ARIMA-forecast until 2040 indicates the preservation of low absolute levels of mortality from hypertension, while high variability of long-term effects is established (Table S2).

Mortality from CIHD in both groups was characterized by pronounced temporal variability throughout the entire period 1952–2022. In the exposed group, the level of mortality from CIHD in certain periods (1962–1972) was statistically significantly higher than the indicators of the comparison group; however, in the future, the differences between the groups were of an unstable character, without the formation of a permanent gap between the curves. ARIMA-modeling showed that the time series of mortality from CIHD does not demonstrate a stable trend (Table S1).

Mortality from AMI in both groups during the period 1952–2022 was characterized by pronounced temporal variability without a stable trend. During the initial period of the study (from 1962 to 1972), statistically significantly higher mortality rates were noted in the exposed group (*p* < 0.001) (Table S1). The forecast until 2040 indicates the preservation of unstable levels of mortality in both groups without the formation of a long-term trend. Wide 95% confidence intervals of forecast estimates reflect high uncertainty of long-term forecasting (Table S2).

Mortality from CCVD in both groups during the period 1952–2022 remained low, with a predominance of zero or single values at most time points. In the exposed group, mortality from CCVD was higher and was characterized by separate periods of sharp increase, in particular, starting from the end of the 1970s, which probably reflects both the accumulated vascular risk and the peculiarities of the coding of the causes of death (Figure 2, Table S1). From 1972 to 2002, the differences in study groups were statistically significant. In the comparison group, cases of death from CCVD were registered episodically and did not form stable temporal dynamics. ARIMA modeling showed that the time series of mortality from CCVD does not demonstrate a stable trend. The forecast until 2040 indicates the maintenance of very low average levels of mortality, while the 95% confidence intervals remain wide, which reflects the extremely high uncertainty of long-term estimates (Table S2).

An analysis of ischemic stroke mortality dynamics in the study groups for the period 1952–2022 revealed temporal variability in rates without the emergence of a stable trend. Intensive mortality rates calculated per 100,000 population ranged from 9.7 to 99.8 per 100,000 population. In the exposed group, ischemic stroke mortality rates from 1962 to the late 1980s were statistically significantly higher compared to the unexposed study group. In the following decades, the differences between the groups leveled out and were characterized by pronounced instability (Figure 2, Table S1).

An analysis of haemorrhagic stroke mortality dynamics in the study groups for the period 1952–2022 revealed that this cause of death is characterized by significant variability. Mortality rates varied widely, from 10.9 to 140.9 per 100,000 population. In the exposed group, haemorrhagic stroke mortality was statistically significantly higher than in the comparison group throughout almost the entire study period up until 2007 (Figure 2, Table S1). ARIMA modeling revealed that the haemorrhagic stroke mortality time series do not exhibit a consistent deterministic trend. For both groups, the adequacy of the models was confirmed by the absence of autocorrelation in the residuals (Ljung–Box test, *p* > 0.05), indicating an accurate description of the random component of the time series (Table S2).

A comparative analysis showed that mortality rates from all types of strokes were statistically significantly higher in the exposed group from the start of the study to 2007 than in the comparison group, with the exception of the late 1990s. Overall, the mortality dynamics were similar to those from haemorrhagic stroke (Figure 2, Table S1). The ARIMA forecast to 2040 indicates continued fluctuation in stroke mortality rates in both groups without the formation of a stable long-term trend (Table S2). Mortality rates from congenital heart disease in the study groups during the study period were characterized by extremely low rates, ranging from 0 to 19.6 per 100,000 population, reflecting the rarity of this cause of death in the overall structure of CVD mortality. Statistically significant differences in mortality rates, with rates in the exposed group exceeding those in the comparison group, were observed only between 1997 and 2002 (Figure 2, Table S1).

### 3.5. Evaluation of the Dose–Response Relationship for CVD Mortality in Study Groups

**Figure 3:**
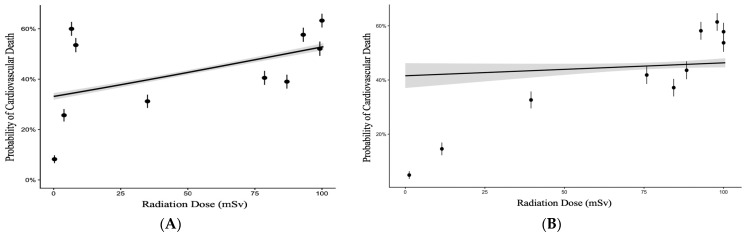
Dose–response relationship for mortality from CVDs (**A**) adjusted for age (**B**). The *x*-axis represents the individual radiation dose (mSv), and the *y*-axis represents the probability of death from cardiovascular diseases (%). Black dots represent the observed CV mortality rates in dose distribution deciles, and vertical lines represent the 95% CI. The solid line represents the predicted probability of death obtained using binary logistic regression, and the gray area corresponds to the 95% CI of the model.

To assess the relationship between cardiovascular mortality and radiation dose, we performed a linear regression analysis (Figure 3). According to the model, the probability of cardiovascular mortality gradually increases with increasing radiation dose. Thus, an increase in dose by 10 mSv (1cSv) is associated with an increase in the odds of death from cardiovascular disease by approximately 8.5% (OR = 1.085; 95% CI 1.075–1.094; *p* < 0.001). Despite the pronounced variability of the observed values between individual dose deciles, the overall trend is characterized by a steady increase in the probability of death with increasing radiation exposure. The confidence interval around the regression line remains relatively narrow, indicating high statistical accuracy of the association estimate (Figure 3A).

Given that cardiovascular mortality is directly related to age, we adjusted the regression model for the effect of radiation dose on cardiovascular mortality in the radiation-exposed population, adjusting for age (Figure 3). Among residents of exposed areas, age turned out to be an independent and most significant predictor of death from cardiovascular diseases (β = 0.0439; *p* < 0.001). Each additional 10 years of age was associated with an increase in the odds of cardiovascular death by approximately 55% (OR = 1.55). Radiation dose demonstrated a positive, but statistically insignificant, association with cardiovascular mortality (β = 0.00194; *p* = 0.097). Each additional 10 mSv (1 cSv) was associated with an increase in the odds of death by approximately 2%, but this relationship did not reach the level of statistical significance (Figure 3B).

**Figure 4:**
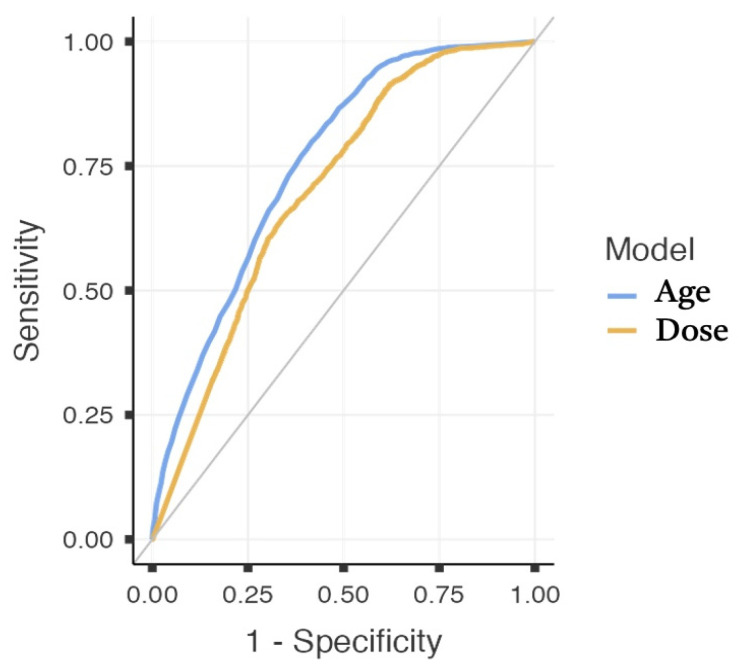
ROC analysis of the dependence of mortality from CVDs on dose and age.

We conducted an ROC analysis of the predictive ability of the radiation dose model in predicting mortality from CVDs (Figure 4). The area under the curve (AUC) for dose was 0.7 (0.689–0.711, *p* < 0.001), which corresponds to satisfactory discriminatory ability of this indicator in relation to mortality from CVDs. At the same time, age was also characterized by high predictive ability, and AUC was 0.755 (0.745–0.765, *p* < 0.001).

## 4. Discussion

The results of our study revealed elevated ASMRs from CVDs in populations living in radioecologically unfavorable conditions for almost the entire study period. The most pronounced differences were observed during the period of surface and airborne nuclear tests and the first decade of underground explosions when relative risks were in the range 2.21–5.31. Only after 2005 did the ASRs in the study groups become equal. Until 1970, there was an increase in ASR in the exposed group of 31.5 cases per year; then, the rates began to slowly decline. This peak of relative mortality risks corresponded to the period when air and ground nuclear weapons testing ceased, plus several years when the rates remained high. It is important to note that the demographic structure of the population has undergone significant changes due to the natural losses of older age groups exposed to direct radiation and the gradual increase in the proportion of their descendants. Furthermore, changes in population composition were associated with migration processes, particularly pronounced in the 1990s after the collapse of the USSR, when large groups of people migrated to the Russian Federation and Germany.

The highest relative risks of mortality over the entire observation period were found for CVDs in general (RR = 1.41), stroke (RR = 2.0), CCVD (RR = 7.88), and congenital heart defects (RR = 2.39). The analysis of the YLL corresponds to the mortality rates from CVDs. The maximum number of YLL was found for AMI (6097.0 in the exposed group versus 6097.0 in the comparison group), hemorrhagic strokes (3857.5 versus 1996.9), and CHD (2696.6 versus 957.7); the differences in the study groups were statistically significant.

Statistically significant differences were found between the study groups in the structure of cardiovascular mortality in terms of CIHD, hemorrhagic stroke, and CCVD. Among study participants born before 1962, CCVD and hemorrhagic stroke were more frequently recorded in the study group. Among people born after the cessation of above-ground nuclear testing, the study group continued to have a higher proportion of deaths from CIHD and hemorrhagic stroke. The mortality rate from CHD remained statistically significantly higher for individuals born after 1990.

In characterizing the dynamics of mortality rates throughout virtually the entire study period, the exposed group showed higher rates compared with the control. Thus, mortality from AMI in the exposed group was statistically significantly higher during the initial study period from 1960 to 1972. Mortality rates from chronic cerebrovascular disease in the exposed group were statistically significantly higher from 1972 to 2002, mortality rates from ischemic stroke were higher from the early 1960s to the late 1980s, and mortality rates from hemorrhagic stroke were statistically significantly higher than those of the comparison group throughout the entire study period. A similar trend was observed for CHD, with the exception of certain short periods when no differences were observed between the study groups. No significant trends in mortality rates over time were observed for hypertension and CIHD across the study groups.

Taken together, the results confirm that the analysis of separate nosological forms does not always adequately reflect the scale of intergroup differences, while the total mortality from CVDs remains the most stable and informative indicator of adverse long-term effects.

Predicting mortality from CVDs and separate forms of CVDs using an ARIMA model did not reveal stable mortality rates across study groups. This is evidenced by the relatively wide confidence intervals, demonstrating the predictive uncertainty and instability of these trends.

Linear regression analysis showed that the probability of cardiovascular mortality gradually increases with increasing radiation dose. An increase in dose of 1 cSv is associated with an increase in the probability of death by approximately 8.5%. The statistical accuracy of this relationship is confirmed by the relatively narrow confidence intervals.

It is well known that numerous risk factors play a role in the development of cardiovascular pathology, the most significant of which are age, obesity, smoking, dyslipidemia, diabetes mellitus, and others. Given that the mortality subregister included only data from medical death certificates, we were unable to conduct a multivariate assessment of the relationship between radiation dose and traditional cardiovascular mortality risk factors other than age. When adjusted for age, it was found to be an independent and significant predictor of death. Each additional 10 years of age was associated with an approximately 55% increase in the likelihood of cardiovascular death. When these two risk factors were assessed jointly, radiation dose demonstrated a positive, but statistically insignificant, association with cardiovascular mortality.

Of the 456 nuclear tests conducted during the entire period of SNTS activity, only 11 tests were able to cause significant radiation exposure (more than 5 mSv during the first year after the test) to the population living in radiation-contaminated areas around the test site. The tasks of the scientists who assessed the radiation doses of the affected population included dosimetry studies aimed at analyzing the contribution of external and internal doses of ionizing radiation. On the example of the village of Dolon, which is located near the territory of the former nuclear test site and suffered mainly from the test on August 29, 1949, it was established that the reconstruction of doses by the calculation method, carried out in six different studies, showed the range of external radiation doses from 0.72 Gy to 2.4 Gy, while the effective doses of radiation varied from 1.1 to 2.2 Sv). The total average dose to the thyroid gland was 1060 mGy, while the maximum contribution to the dose was made by the consumption of dairy products containing I131 (1050 mGy), with a total external radiation dose of 1240 mGy. The power of the internal dose of radiation increases significantly with the increase in the number of particles with a diameter of less than 50 microns [13]. However, the number of studies aimed at estimating internal radiation doses of the radiation-exposed population of the Semipalatinsk region is extremely limited, since such studies are associated with the need to accurately estimate the consumption of various dairy, meat and other agricultural products produced locally. It is very difficult to conduct such a retrospective assessment more than 70 years after the beginning of nuclear tests.

Possible biological mechanisms of radiation-induced cardiovascular diseases are proposed in some studies [29,30,31,32]. Among the possible mechanisms, the most likely is the inflammatory response, especially at high doses of radiation, as well as the possible role of the effect of radiation on the immune system. Oxidative stress, proinflammatory and prothrombotic reactions, in which cytokines and transcription factors participate, can play a role in radiation-induced endothelial dysfunction, leading to atherosclerosis [33]. Macrovascular and microvascular effects in cardiac and extracardiac structures are important, but additional radiobiological studies are necessary [34]. At lower doses of ionizing radiation, the radiobiological mechanism is much less studied. Several mechanisms have been proposed, for example, the death of monocytes in the arterial intima [35], as well as radiation-induced senescence of endothelial cells and the associated adhesion of monocytes [36,37,38,39]. In addition to epidemiological studies of the effects of low doses, cellular and tissue reaction to fractionated or chronic radiation requires more careful study.

Radiation-induced endothelial damage is a common pathogenetic mechanism for both organs, but its consequences manifest themselves differently. In the brain, endothelial damage can lead to disruption of the blood–brain barrier, neurovascular dysfunction, altered microvascular permeability, inflammatory activation, and secondary damage to nervous tissue. In the heart, radiation-induced vascular damage is more often viewed through the lens of endothelial dysfunction, microcirculatory disorders, accelerated atherogenesis, myocardial ischemia, and subsequent remodeling. Thus, radiation-induced vascular pathology in the brain and heart share a common endothelial component, but organ-specific manifestations are determined by differences in the structure and function of the respective vascular beds [40,41]. A deeper understanding of the biological mechanisms underlying radiation-induced damage to the heart tissue, cardiovascular system, and blood vessels is important for preventing mortality from cardiovascular diseases among the population exposed to nuclear tests at the Semipalatinsk test site.

Of considerable interest to us was the possibility of comparing our results with the results of similar studies related to the assessment of cardiovascular mortality in individuals exposed to radiation situations. Of particular interest to us were the results of studies among the atomic bomb survivors in Japan due to the comparability of the irradiation conditions and the duration of the study. The results obtained in the cohort Life Span Study (LSS) revealed a number of important conclusions regarding the long-term consequences of exposure to ionizing radiation, in terms of the development of excess mortality from cardiovascular diseases [42,43]. Thus, in the Adult Health Study (AHS) cohort, a subcohort of the Life Span Study, mortality from CVDs, as in our study, was primarily due to stroke. Mortality increased from the early 1950s to the 1980s and then gradually declined; the authors attributed this to improved stroke diagnostic methods, which facilitated earlier and more effective treatment. Hypertension mortality rates among exposed individuals were similar to those in the general population, peaking in the 1970s. Hemorrhagic stroke mortality rates in the Japanese cohort persisted until the 1990s, gradually declining in subsequent years. The highest excess relative risks were found for heart diseases in general (ERR/Gy = 0.14), stroke (ERR/Gy = 0.16), hypertension (ERR/Gy = 0.37), and chronic heart failure (ERR/Gy = 0.22) [44,45]. However, the magnitude of the risk at doses less than 0.5 Gy remains poorly defined [42].

The risk of mortality from myocardial infarction and hypertension in individuals exposed to radiation at the age of under 40 years increased depending on the radiation dose; no linear relationship was found for other nosologies [43]. Analysis of the results of Japanese studies revealed similar trends regarding the leading causes of mortality in the structure of CVDs, as well as the values of relative mortality rates, with those in our study. Regarding the dynamics of mortality rates, in our study, they remained more stable throughout the study, despite the presence of individual peaks in rates, which can be explained by the prolonged period of combined external and internal irradiation of the affected population of Kazakhstan at higher doses, whereas for members of the Japanese cohort, the average radiation dose was 19 times lower (38 ± 58 mGy) [43].

Japanese scientists have found that the excess risk of mortality from CVDs depends on age at the time of exposure: with increasing age, a tendency toward a decrease in ERR is observed; however, no dependence of ERR on gender or the period from the onset of radiation exposure was found [42,43]. Of interest is the fact that adjustment for traditional risk factors for CVDs, such as bad habits, education level, obesity, and diabetes, reduced the relative risk of mortality from all CVDs, stroke, or heart disease associated with exposure to ionizing radiation by less than 20%.

Of particular interest are the data demonstrating a link between radiation exposure and subclinical cardiovascular damage among survivors in the LSS population. These results explain the high mortality rates in the studied population. In particular, irradiated individuals exhibited elevated systolic and diastolic blood pressure, as well as changes in lipid metabolism, manifested by the accelerated development of atherogenic dyslipidemia [46,47,48]. Such metabolic disturbances may be a possible mechanism linking radiation exposure to the development of a number of pathological conditions, including fatty liver disease, aortic arch calcification, hypertension, and coronary heart disease [49]. Additional experimental and clinical data indicate that ionizing radiation may contribute to the development of atherosclerotic processes by enhancing the systemic inflammatory response. Thus, it has been established that at a dose of approximately 1 Gy, the level of C-reactive protein increases by approximately 28%, while the concentration of interleukin-6 increases by approximately 9%, which indicates a possible role of chronic inflammation in the pathogenesis of radiation-induced cardiovascular disorders [50,51].

An analysis of mortality rates from CVDs among residents of settlements located around the SNTS was preliminarily conducted on the historical Semipalatinsk cohort, which included 19,545 residents of Kazakhstan exposed to radioactive fallout in the medium and low dose range (from 0 to 630 mGy to the whole body). The cohort study was conducted from 1960 to 1990. Mortality rates from CVDs in the exposed cohort significantly exceeded those in the unexposed cohort. The relative risk for all CVDs, adjusted for radiation dose, was 2.27 (95% CI: 2.10; 2.45), for heart disease was 2.23 (95% CI: 2.02; 2.46) and for stroke was 2.30 (2.00; 2.65). However, the dose–response relationship was not statistically significant for either all cardiovascular diseases or for heart disease or stroke [19].

Another study of radiation-related cardiovascular mortality in the South Ural Techa River cohort with an observation period of 1950–2003 found that the excess relative risk per 100 mGy for all circulatory diseases was 3.6%, and for coronary heart disease, 5.6%. No evidence was found for an increased risk of mortality from cerebrovascular diseases [52]. The differences in the results of this study and ours are likely due to different exposure conditions, since the population living along the Techa River was exposed to a higher degree of internal radiation from 90Sr, 89Sr, and 137Cs isotopes as a result of water discharges from the Mayak nuclear power plant.

An analysis of the literature reveals a wide variety of radiation situations that can influence morbidity and mortality from CVDs in various population groups. These situations primarily include occupational exposure in cohorts of uranium miners, nuclear industry workers, and Chernobyl disaster liquidators [53,54,55,56]. The advantages of these cohort studies include careful long-term follow-up, systematic and regular medical examinations, accurate recording of adverse events, and calculation of individual radiation doses. However, studies including residents of radioecologically unfavorable areas have been extremely rare due to the unique nature of each of these situations. Conducting cohort studies in this context is significantly complicated by migration processes, changes in demographic structure, and significant difficulties in calculating radiation doses and conducting medical examinations. Therefore, calculating excess mortality risks per unit of radiation dose is impossible.

### Limitations of the Study

The studied population was characterized by long-term residence in radiation-contaminated areas, which resulted in repeated external and internal exposure to a wide range of doses until the cessation of nuclear testing in 1989. The study had a cross-sectional design, although a cohort study would have been the most appropriate design. Other limitations of our study include the inconsistency of medical examinations and the incomplete coverage of the entire population, which could have led to inaccuracies in the coding of causes of death. Furthermore, until the early 1960s, cause-of-death coding was often performed by mid-level medical professionals. It was only when the first doctors, graduates of the Semipalatinsk Medical Institute, arrived in the districts that the process of diagnosing and coding diseases improved significantly. We extracted information for our cause-of-death analysis from the SSAMR, which was only established in 2002 and was therefore retrospective. It should be noted that access to medical care among the Kazakhstani residents we studied was quite low before the early 1960s, and diagnostic capabilities were limited.

The present study is based on retrospective registry data collected over a long observation period. Death certificates contained information on the patient’s age, education, marital status, and diagnosis. Information on several important cardiovascular risk factors, including smoking, alcohol consumption, hypertension, diabetes mellitus, obesity, dietary habits, socioeconomic conditions, and access to medical care, was not available and therefore could not be included in the analysis. Consequently, residual confounding cannot be excluded. In addition, radiation doses were reconstructed retrospectively, and the relative contributions of external irradiation and internal radionuclide incorporation could not be evaluated separately. These limitations should be considered when interpreting the observed associations between radiation exposure and cardiovascular mortality.

## 5. Conclusions

An assessment of mortality dynamics and predictions was conducted, as well as the number of years of life lost due to CVDs in Kazakhstani residents living in areas exposed to radioactive contamination. The highest relative mortality risks were found for CVDs in general, stroke, chronic cerebrovascular disease, and congenital heart defects. The highest YLLs were observed for acute myocardial infarction, hemorrhagic stroke, and congenital heart disease; differences between study groups were statistically significant. Linear regression analysis showed that the probability of cardiovascular mortality gradually increases with increasing radiation dose. Age was found to be an independent and significant predictor of cardiovascular death. The predictive model using ROC analysis showed good discriminatory ability for both radiation dose and age.

Despite significant changes in the demographic structure of the study groups over the long study period, mortality rates from CVDs in the exposed group remained higher than those in the control group.

## Figures and Tables

**Table 1:** Key radionuclides resulting from radioactive fallout from nuclear tests.

Radionuclide	Half Life	Principal Radiation	Target Organ/Biological Effect
Iodine-131	≈8 days	β, γ	Concentrates in the thyroid gland; irradiation of thyroid tissue may increase the risk of thyroid cancer, particularly after childhood exposure
Strontium-90	≈28.8 years	β	A chemical analog of calcium. Deposits in bone and bone marrow; prolonged irradiation may increase the risk of leukemia and bone cancer.
Cesium-137	≈30 years	β, γ	Distributed relatively uniformly throughout soft tissues and contributes to whole-body irradiation.
Plutonium-239	24,100 years	α	Inhaled plutonium may be retained in the lungs; a fraction may translocate to the liver and skeleton, increasing long-term cancer risk.
Plutonium-240	6560 years	α	Accumulates primarily in the skeleton and liver; alpha irradiation may increase the risk of bone and liver cancers.
Americium-241	≈432 years	α, γ	Deposits mainly in bone and liver; long-term alpha irradiation may increase the risk of bone and liver cancers.

**Table 2:** Age-standardized mortality rates from CVDs in study groups by study periods.

Study Period	Study Group ASMR (95% CI)	Control Group ASMR (95% CI)	RR (95% CI)
1949–1954	1614.8 (1455.4–1774.2)	731.4 (618.2–844.7)	2.21 (1.84–2.65)
1954–1959	1209.8 (1079.3–1340.3)	353 (279.8–426.3)	3.43 (2.71–4.33)
1960–1964	2104.8 (1945.2–2264.5)	396.4 (320.2–472.6)	5.31 (4.32–6.53)
1965–1969	1841 (1700.4–1981.5)	538.6 (453.1–624)	3.42 (2.87–4.08)
1970–1974	2134.5 (1986.6–2282.4)	582.8 (496.4–669.2)	3.66 (3.11–4.31)
1975–1979	2058.6 (1912.7–2204.5)	825.1 (724.3–926)	2.49 (2.17–2.87)
1980–1984	1725.8 (1598.6–1852.9)	1217.7 (1100.1–1335.4)	1.42 (1.26–1.6)
1985–1989	1532 (1409.9–1654.1)	938.9 (838.4–1039.5)	1.63 (1.43–1.86)
1990–1994	1414.8 (1300.2–1529.3)	1097.8 (989.7–1205.9)	1.29 (1.13–1.46)
1995–1999	1318.1 (1209.9–1426.2)	977.4 (883.5–1071.4)	1.35 (1.19–1.53)
2000–2004	1601.6 (1475.5–1727.7)	986.5 (875.9–1097.1)	1.62 (1.42–1.86)
2005–2009	1640.9 (1509.6–1772.2)	1897.7 (1695.7–2099.7)	0.86 (0.76–0.99)
2010–2014	1026.7 (921.7–1131.8)	1008.2 (858–1158.4)	1.02 (0.85–1.22)
2015–2019	844.1 (744.7–943.5)	915.5 (768.6–1062.4)	0.92 (0.76–1.13)
After 2020	1507.5 (1366.6–1648.4)	1381.3 (1185–1577.6)	1.09 (0.92–1.29)

**Table 3:** Mortality rates (per 100,000 population) and relative risks (RRs) for different types of CVDs in the study groups over the entire observation period.

Cause of Death	Study Group		Control Group		RR (95% CI)
	N	Mortality Rate per 100,000 (95% CI)	N	Mortality Rate per 100,000 (95% CI)	
CVDs	3482	612.9 (592.7–633.6)	1886	435.8 (416.3–455.9)	1.41 (1.33–1.49)
Hypertension	200	35.2 (30.5–40.4)	124	28.7 (23.8–34.2)	1.23 (0.98–1.54)
Chronic ischemic heart disease	638	112.3 (103.8–121.4)	1022	236.2 (221.9–251.1)	0.48 (0.43–0.52)
Acute myocardial infarction	584	102.8 (94.6–111.5)	346	80.0 (71.7–88.8)	1.29 (1.13–1.47)
Stroke (ischemic + hemorrhagic)	720	126.7 (117.6–136.3)	274	63.3 (56.0–71.3)	2.00 (1.74–2.30)
Chronic cerebrovascular diseases	207	36.4 (31.6–41.8)	20	4.6 (2.8–7.1)	7.88 (4.98–12.48)
Congenital heart defects	44	7.7 (5.6–10.4)	14	3.2 (1.8–5.4)	2.39 (1.31–4.37)

Mortality rates are presented per 100,000 population, and 95% confidence intervals (95% CIs) for mortality rates are calculated using a Poisson distribution.

**Table 4:** Structure of mortality from CVDs in study groups depending on the period of birth.

Cause of Death	Study Group, N (%)			*p*	Control Group, N (%)			*p*	*p* **
	Before 1962	1963–1989	After 1990		Before 1962	1963–1989	After 1990		
Hypertension	196 (5.8%)	3 (2.2%)	0 (0.0%)	0.106	98 (6.2%)	2 (2.9%)	0 (0.0%)	0.33	1 = 0.621 2 = 0.755 3 = 1
CIHD	1033 (30.8%)	32 (23.0%)	0 (0.0%)	0.002 0.008 *	593 (37.6%)	5 (7.1%)	0 (0.0%)	<0.001 <0.001 *	1 < 0.001 2 = 0.004 3 = 1
AMI	572 (17.1%)	15 (10.8%)	0 (0.0%)	0.024 0.005 *	280 (17.7%)	29 (41.4%)	4 (30.8%)	<0.001 <0.001 *	1 = 0.566 2 < 0.001
CCVD	218 (6.5%)	10 (7.2%)	2 (11.1%)	0.69	21 (1.3%)	1 (1.4%)	3 (23.1%)	<0.001 <0.001 *	1 < 0.001 2 = 0.078 3 = 0.371
Ischemic stroke	302 (9.0%)	8 (5.8%)	0 (0.0%)	0.172	128 (8.1%)	1 (1.4%)	0 (0.0%)	0.07	1 = 0.293 2 = 0.146 3 = 1
Hemorrhagic stroke	392 (11.7%)	17 (12.2%)	0 (0.0%)	0.29	95 (6.0%)	1 (1.4%)	0 (0.0%)	0.18	1 < 0.001 2 = 0.009 3 = 1
CHD	8 (0.2%)	22 (15.8%)	12 (66.7%)	<0.001 <0.001 *	2 (0.1%)	10 (14.3%)	1 (7.7%)	<0.001 <0.001 *	1 = 0.415 2 = 0.77 3 = 0.001

For comparison, χ^2^ with Benjamini–Hochberg correction was used. *, ** comparison of the main and control groups by periods: 1—before 1962; 2—1963–1989; 3—later 1990; CIHD—Chronic Ischemic Heart Disease; AMI—Acute Myocardial Infarction; CCVD—Chronic Cerebrovascular Disease; CHD—Congenital Heart Disease.

**Table 5:** Characteristics of the YLL rates due to CVDs per 100 thousand population.

Cause of Death	Study Group	Control Group	*p*
Hypertension	1174.2	1154.7	0.578
CIHD	4518.0	5614.0	<0.001
AMI	6097.0	5893.0	<0.001
CCVD	810.0	641.9	0.049
Ischemic stroke	2091.0	1303.9	0.442
Hemorrhagic stroke	3857.5	1996.9	0.0195
CHD	2696.6	957.7	0.001

CIHD—Chronic Ischemic Heart Disease; AMI—Acute Myocardial Infarction; CCVD—Chronic Cerebrovascular Disease; CHD—Congenital Heart Disease.

## Data Availability

The raw data supporting the conclusions of this article will be made available by the authors on request.

## Supplementary Materials

The following supporting information can be downloaded at: https://www.mdpi.com/article/10.3390/ijerph23070874/s1, Table S1: Mortality rates from CVDs and separate diseases in study groups per 100,000 (95% CI); Table S2: ARIMA Forecasting Summary Table.
