# Supplementary material for: Long-Term Trends and Prognosis in Cardiovascular Mortality in the Kazakhstani Population Living Around the Semipalatinsk Nuclear Test Site
Source: Int J Environ Res Public Health. 2026 Jul 5;23(7):874. doi: 10.3390/ijerph23070874 (PMC13412042; doi:10.3390/ijerph23070874)
Supplement: Supplementary file 1 [file ijerph-23-00874-s001.zip › Supplement S1.pdf]

Supplement S1. Mortality rates from CVD and separate diseases in study groups per 100,000 (95% CI)

Cause of death	Year	Exposed group	Unexposed group	p
CVD	1952	153.0 (110.2–206.8)	168.1 (120.6–228.0)	0,742
	1957	145.7 (106.2–194.9)	107.0 (71.7–153.7)	0,202
	1962	642.7 (560.2–733.9)	73.9 (45.7–112.9)	<0.001
	1967	641.8 (563.0–728.5)	202.0 (154.8–258.9)	<0.001
	1972	701.8 (621.1–790.0)	303.1 (245.8–369.8)	<0.001
	1977	843.0 (755.6–937.9)	427.7 (359.6–505.0)	<0.001
	1982	773.7 (694.0–860.1)	655.5 (574.4–744.9)	0,054
	1987	648.2 (574.2–729.0)	466.4 (400.2–540.4)	<0.001
	1992	647.5 (575.1–726.4)	519.3 (452.0–593.7)	0,014
	1997	532.3 (467.7–603.3)	577.1 (509.6–651.1)	0,375
	2002	741.6 (660.4–829.9)	485.7 (412.5–568.2)	<0.001
	2007	868.2 (776.4–968.0)	981.3 (843.2–1135.6)	0,201
	2012	469.3 (401.7–544.9)	445.3 (351.4–556.5)	0,735
	2017	420.6 (355.0–495.0)	592.1 (476.7–727.0)	0,011
	2022	702.9 (614.1–801.1)	748.1 (610.6–907.2)	0,589
Hypertension	1952	0.0 (0.0–13.4)	0.0 (0.0–15.1)	1
	1957	0.0 (0.0–11.9)	11.1 (2.3–32.4)	0,102
	1962	14.7 (4.8–34.4)	14.1 (3.8–36.0)	1
	1967	21.5 (9.3–42.3)	0.0 (0.0–12.0)	0,01
	1972	30.7 (15.9–53.7)	9.4 (1.9–27.4)	0,068
	1977	24.9 (12.0–45.9)	18.5 (6.8–40.2)	0,623
	1982	2.3 (0.1–12.6)	25.1 (11.5–47.7)	0,07
	1987	9.3 (2.5–23.9)	21.1 (9.1–41.5)	0,248
	1992	8.9 (2.4–22.9)	41.3 (24.0–66.0)	0,003
	1997	26.1 (13.5–45.5)	54.6 (35.4–80.7)	0,033
	2002	56.3 (35.7–84.5)	43.6 (23.8–73.1)	0,51
	2007	106.9 (76.3–145.5)	49.1 (22.4–93.1)	0,033
	2012	60.0 (37.6–90.9)	28.9 (9.4–67.5)	0,152
	2017	69.6 (44.6–103.6)	58.6 (26.8–111.2)	0,851
	2022	109.3 (76.2–152.1)	87.2 (45.0–152.2)	0,633
AMI	1952	10.9 (2.3–31.9)	45.1 (22.5–80.7)	0,029
	1957	22.7 (9.1–46.7)	14.8 (4.0–37.8)	0,558
	1962	135.6 (99.3–180.9)	3.5 (0.1–19.6)	<0.001
	1967	153.1 (115.9–198.3)	19.5 (7.2–42.5)	<0.001
	1972	158.8 (121.8–203.6)	46.9 (26.2–77.3)	<0.001
	1977	124.7 (92.6–164.4)	107.7 (75.0–149.8)	0,515
	1982	97.0 (70.2–130.7)	150.6 (113.2–196.5)	0,032
	1987	104.9 (76.5–140.4)	6 3.2 (40.5–94.1)	0,053
	1992	145.1 (112.0–185.0)	84.9 (59.2–118.1)	0,012
	1997	134.7 (103.3–172.7)	70.0 (47.8–98.8)	0,003
	2002	137.1 (103.5–178.0)	158.8 (118.2–208.8)	0,496
	2007	144.3 (108.4–188.2)	218.1 (155.8–296.9)	0,049
	2012	30.0 (15.0–53.7)	57.8 (27.7–106.3)	0,159

	2017	31.9 (15.9–57.1)	104.1 (59.5–169.1)	0,003
	2022	37.5 (19.4–65.5)	87.2 (45.0–152.2)	0,044
CIHD	1952	83.8 (53.1–125.7)	77.9 (46.9–121.6)	0,878
	1957	71.2 (44.6–107.8)	36.9 (17.7–67.9)	0,109
	1962	283.0 (229.3–345.6)	38.7 (19.3–69.2)	<0.001
	1967	257.8 (208.8–314.8)	133.6 (95.8–181.2)	<0.001
	1972	25.6 (12.3–47.1)	156.3 (116.0–206.0)	<0.001
	1977	37.4 (20.9–61.7)	200.0 (154.4–254.9)	<0.001
	1982	36.1 (20.6–58.6)	326.4 (269.9–391.1)	<0.001
	1987	30.3 (16.1–51.8)	289.9 (238.2–349.4)	<0.001
	1992	24.6 (12.3–43.9)	327.6 (274.7–387.7)	<0.001
	1997	171.6 (135.9–213.9)	354.1 (301.7–413.1)	<0.001
	2002	31.8 (16.9–54.4)	208.6 (161.7–264.9)	<0.001
	2007	37.4 (20.4–62.8)	588.8 (483.0–710.9)	<0.001
	2012	212.8 (168.2–265.6)	150.3 (98.2–220.3)	0,141
	2017	174.1 (132.8–224.0)	286.3 (208.0–384.3)	0,014
	2022	287.4 (231.7–352.5)	414.0 (313.5–536.4)	0,032
CCVD	1952	0.0 (0.0–13.4)	4.1 (0.1–22.8)	0,47
	1957	0.0 (0.0–11.9)	0.0 (0.0–13.6)	1
	1962	2.9 (0.1–16.4)	0.0 (0.0–13.0)	1
	1967	2.7 (0.1–15.0)	0.0 (0.0–12.0)	1
	1972	7.7 (1.6–22.5)	3.1 (0.1–17.4)	0,632
	1977	64.8 (42.4–95.0)	6.2 (0.7–22.2)	<0.001
	1982	94.7 (68.3–128.1)	16.7 (6.1–36.4)	<0.001
	1987	21.0 (9.6–39.8)	2.6 (0.1–14.7)	0,024
	1992	38.0 (22.1–60.8)	0.0 (0.0–9.0)	<0.001
	1997	50.0 (31.7–75.0)	0.0 (0.0–8.1)	<0.001
	2002	22.0 (10.1–41.8)	0.0 (0.0–11.5)	0,006
	2007	18.7 (7.5–38.5)	16.4 (3.4–47.8)	1
	2012	35.5 (18.9–60.6)	34.7 (12.7–75.5)	1
	2017	34.8 (18.0–60.8)	0.0 (0.0–24.0)	0,023
	2022	137.5 (99.9–184.5)	0.0 (0.0–26.8)	<0.001
Ischemic stroke	1952	10.9 (2.3–31.9)	20.5 (6.7–47.8)	0,488
	1957	9.7 (2.0–28.4)	3.7 (0.1–20.6)	0,628
	1962	76.7 (50.1–112.3)	7.0 (0.9–25.4)	<0.001
	1967	77.9 (52.2–111.8)	19.5 (7.2–42.5)	<0.001
	1972	69.2 (45.6–100.6)	34.4 (17.2–61.5)	0,051
	1977	99.8 (71.3–135.9)	46.2 (25.8–76.1)	0,01
	1982	79.0 (55.0–109.8)	41.8 (23.4–69.0)	0,045
	1987	60.6 (39.6–88.8)	31.6 (16.3–55.2)	0,073
	1992	53.6 (34.3–79.7)	36.4 (20.4–60.0)	0,264
	1997	34.8 (19.9–56.5)	48.1 (30.1–72.8)	0,335
	2002	41.6 (24.2–66.6)	31.1 (14.9–57.3)	0,563
	2007	61.4 (39.0–92.2)	49.1 (22.4–93.1)	0,707
	2012	27.3 (13.1–50.2)	63.6 (31.8–113.8)	0,06
	2017	26.1 (11.9–49.6)	91.1 (49.8–152.8)	0,003
	2022	50.0 (28.6–81.2)	65.4 (29.9–124.1)	0,517

Hemorrhagic stroke	1952	10.9 (2.3–31.9)	4.1 (0.1–22.8)	0,628
	1957	3.2 (0.1–18.0)	22.1 (8.1–48.2)	0,056
	1962	97.3 (67.0–136.6)	7.0 (0.9–25.4)	<0.001
	1967	99.4 (70.0–136.9)	16.3 (5.3–38.0)	<0.001
	1972	140.9 (106.1–183.4)	46.9 (26.2–77.3)	<0.001
	1977	87.3 (60.8–121.4)	15.4 (5.0–35.9)	<0.001
	1982	56.4 (36.5–83.3)	30.7 (15.3–54.9)	0,095
	1987	58.3 (37.7–86.0)	15.8 (5.8–34.4)	0,002
	1992	71.4 (48.9–100.9)	7.3 (1.5–21.3)	<0.001
	1997	73.9 (51.2–103.2)	41.5 (25.0–64.9)	0,053
	2002	88.1 (61.7–122.0)	40.5 (21.6–69.2)	0,014
	2007	104.2 (74.1–142.4)	27.3 (8.9–63.6)	0,002
	2012	54.6 (33.3–84.3)	52.0 (23.8–98.8)	1
	2017	75.4 (49.3–110.5)	45.5 (18.3–93.8)	0,263
	2022	46.9 (26.2–77.3)	72.6 (34.8–133.6)	0,281
All strokes	1952	21.9 (8.0–47.6)	24.6 (9.0–53.5)	1
	1957	12.9 (3.5–33.1)	25.8 (10.4–53.2)	0,367
	1962	173.9 (132.4–224.4)	14.1 (3.8–36.0)	<0.001
	1967	177.2 (137.1–225.5)	35.8 (17.9–64.1)	<0.001
	1972	210.0 (167.0–260.7)	81.3 (53.1–119.1)	<0.001
	1977	187.1 (147.1–234.5)	61.5 (37.6–95.0)	<0.001
	1982	135.3 (103.3–174.2)	72.5 (47.4–106.3)	0,007
	1987	118.9 (88.5–156.4)	47.4 (28.1–75.0)	<0.001
	1992	125.0 (94.4–162.4)	43.7 (25.9–69.0)	<0.001
	1997	108.6 (80.6–143.2)	89.6 (64.3–121.6)	0,402
	2002	129.7 (97.2–169.7)	71.6 (45.4–107.4)	0,015
	2007	165.6 (127.0–212.3)	76.3 (41.7–128.1)	0,007
	2012	81.8 (55.2–116.8)	115.7 (70.6–178.6)	0,229
	2017	101.5 (70.7–141.2)	136.6 (84.6–208.9)	0,311
	2022	96.9 (65.8–137.5)	138.0 (83.1–215.5)	0,220
CHD	1952	7.3 (0.9–26.3)	8.2 (1.0–29.6)	1
	1957	6.5 (0.8–23.4)	0.0 (0.0–13.6)	0,502
	1962	5.9 (0.7–21.3)	0.0 (0.0–13.0)	0,504
	1967	5.4 (0.7–19.4)	0.0 (0.0–12.0)	0,505
	1972	7.7 (1.6–22.5)	0.0 (0.0–11.5)	0,257
	1977	7.5 (1.5–21.9)	6.2 (0.7–22.2)	1
	1982	11.3 (3.7–26.3)	13.9 (4.5–32.5)	0,76
	1987	16.3 (6.6–33.6)	5.3 (0.6–19.0)	0,187
	1992	6.7 (1.4–19.6)	2.4 (0.1–13.5)	0,626
	1997	13.0 (4.8–28.4)	0.0 (0.0–8.1)	0031
	2002	19.6 (8.5–38.6)	0.0 (0.0–11.5)	0,011
	2007	0.0 (0.0–9.9)	10.9 (1.3–39.4)	0,108
	2012	2.7 (0.1–15.2)	0.0 (0.0–21.3)	1
	2017	0.0 (0.0–10.7)	0.0 (0.0–24.0)	1
	2022	0.0 (0.0–11.5)	0.0 (0.0–26.8)	1
