# Supplementary material for: Long-Term Trends and Prognosis in Cardiovascular Mortality in the Kazakhstani Population Living Around the Semipalatinsk Nuclear Test Site
Source: Int J Environ Res Public Health. 2026 Jul 5;23(7):874. doi: 10.3390/ijerph23070874 (PMC13412042; doi:10.3390/ijerph23070874)
Supplement: Supplementary file 1 [file ijerph-23-00874-s001.zip › Supplement S2.pdf]

Supplement S2. ARIMA Forecasting Summary Table

Disease	Year	Forecast in the exposed group	95% CI	Forecast in the unexposed group	95% ДИ
Overall mortality from CVD	2027	706.1	369.7-1042.6	673.2	276.5-1069.9
	2032	600.0	159.0-1041.1	673.2	226.8-1119.6
	2037	600.0	159.0-1041.1	673.2	182.1-1164.3
	2040	600.0	159.0-1041.1	673.2	141.2-1205.2
Hypertension	2027	109.4	12.6–892.4	87.1	7.0–975.0
	2032	109.4	4.7–2123.3	87.1	1.9–2642.0
	2037	109.4	2.0–4128.0	87.1	0.4–5675.4
	2040	109.4	0.7–7229.5	87.1	0.0–10811.9
CIHD	2027	287.4	32.4–2492.7	414.0	112.4–1517.3
	2032	287.4	12.7–6092.6	414.0	65.3–2597.2
	2037	287.4	5.9–12094.5	414.0	42.9–3923.0
	2040	287.4	2.9–21558.4	414.0	30.0–5553.8
AMI	2027	37.5	9.4–141.0	87.2	15.5–469.4
	2032	37.5	5.1–242.8	87.2	7.3–940.3
	2037	37.5	3.0–368.2	87.2	3.8–1601.9
	2040	37.5	1.8–522.8	87.2	2.1–2509.6
CCVD	2027	137.5	21.1–868.2	0.0	0.0–17.8
	2032	137.5	9.3–1859.3	0.0	0.0–62.6
	2037	137.5	4.7–3334.4	0.0	0.0–160.6
	2040	137.5	2.5–5455.5	0.0	0.0–353.9
Ischemic stroke	2027	50.0	13.8–175.1	65.4	20.3–206.0
	2032	50.0	7.8–293.4	65.4	12.3–330.7
	2037	50.0	5.0–435.5	65.4	8.2–475.1
	2040	50.0	3.3–607.5	65.4	5.8–644.8
Hemorrhagic stroke	2027	46.9	6.6–299.8	72.6	12.0–416.3
	2032	46.9	2.6–643.2	72.6	5.3–855.2
	2037	46.9	1.0–1154.5	72.6	2.6–1485.1
	2040	46.9	0.2–1890.0	72.6	1.3–2364.6
CHD	2027	0.0	0.0–6.2	0.0	0.0–12.2
	2032	0.0	0.0–15.2	0.0	0.0–37.4
	2037	0.0	0.0–29.4	0.0	0.0–86.1
	2040	0.0	0.0–50.5	0.0	0.0–172.9
